# CAR-T Therapy for Pediatric High-Grade Gliomas: Peculiarities, Current Investigations and Future Strategies

**DOI:** 10.3389/fimmu.2022.867154

**Published:** 2022-05-04

**Authors:** Laura Antonucci, Gabriele Canciani, Angela Mastronuzzi, Andrea Carai, Giada Del Baldo, Francesca Del Bufalo

**Affiliations:** ^1^ Department of Paediatric Haematology and Oncology, Cell and Gene Therapy, Bambino Gesù Children’s Hospital, IRCCS, Rome, Italy; ^2^ Neurosurgery Unit, Department of Neuroscience and Neurorehabilitation, Bambino Gesù Children’s Hospital, IRCCS, Rome, Italy

**Keywords:** high-grade gliomas (HGG), CAR-T therapies, immunotherapy, tumor microenvironment (TME), next generation CAR-T cells

## Abstract

High-Grade Gliomas (HGG) are among the deadliest malignant tumors of central nervous system (CNS) in pediatrics. Despite aggressive multimodal treatment - including surgical resection, radiotherapy and chemotherapy - long-term prognosis of patients remains dismal with a 5-year survival rate less than 20%. Increased understanding of genetic and epigenetic features of pediatric HGGs (pHGGs) revealed important differences with adult gliomas, which need to be considered in order to identify innovative and more effective therapeutic approaches. Immunotherapy is based on different techniques aimed to redirect the patient own immune system to fight specifically cancer cells. In particular, T-lymphocytes can be genetically modified to express chimeric proteins, known as chimeric antigen receptors (CARs), targeting selected tumor-associated antigens (TAA). Disialoganglioside GD2 (GD-2) and B7-H3 are highly expressed on pHGGs and have been evaluated as possible targets in pediatric clinical trials, in addition to the antigens common to adult glioblastoma – such as interleukin-13 receptor alpha 2 (IL-13α2), human epidermal growth factor receptor 2 (HER-2) and erythropoietin-producing human hepatocellular carcinoma A2 receptor (EphA2). CAR-T therapy has shown promise in preclinical model of pHGGs but failed to achieve the same success obtained for hematological malignancies. Several limitations, including the immunosuppressive tumor microenvironment (TME), the heterogeneity in target antigen expression and the difficulty of accessing the tumor site, impair the efficacy of T-cells. pHGGs display an immunologically cold TME with poor T-cell infiltration and scarce immune surveillance. The secretion of immunosuppressive cytokines (TGF-β, IL-10) and the presence of immune-suppressive cells – like tumor-associated macrophages/microglia (TAMs) and myeloid-derived suppressor cells (MDSCs) - limit the effectiveness of immune system to eradicate tumor cells. Innovative immunotherapeutic strategies are necessary to overcome these hurdles and improve ability of T-cells to eradicate tumor. In this review we describe the distinguishing features of HGGs of the pediatric population and of their TME, with a focus on the most promising CAR-T therapies overcoming these hurdles.

## Pediatric High-Grade Gliomas (pHGGs)

Pediatric HGGs are among the most common malignant brain tumors in pediatrics and represent the leading cause of cancer related death in childhood (1). Traditionally, pediatric and adult gliomas were commonly classified according to the WHO grading, with HGGs including WHO grade III and IV aggressive tumors (2). Over the last years, key differences in epigenomic and genetic features between pHGGs and their adult counterparts emerged, despite analogies in aggressiveness and histology (3). In 2021 WHO Classification of CNS tumors, pediatric gliomas are differentiated from adults– on the heels of 2016 WHO classification – and the term “glioblastoma” is abandoned in pediatric oncologic setting (4). Four groups of pHGGs are now described: *Diffuse midline glioma, H3 K27-altered*; *Diffuse hemispheric glioma, H3 G34-mutant*; *Diffuse pediatric-type high-grade glioma, H3-wildtype and IDH-wildtype*; and *Infant-type hemispheric glioma* (**Table 1**). *Diffuse midline glioma, H3 K27-altered* had already been included in previous classification but the term “altered” aims at including other mechanisms besides previously reported H3-K27 mutations. This group encompasses DIPG, one of the most aggressive types of pHGG. *Diffuse hemispheric gliomas, H3 G34-mutant* typically arise in cerebral hemispheres and are characterized by a G34R/V substitution of histone H3 due to a mutation of H3F3A gene (5). *Diffuse pediatric-type high-grade glioma, H3-wildtype and IDH-wildtype* encompass a heterogeneous group of pHGGs not showing either H3 or IDH mutations. Finally, *Infant-type hemispheric glioma* include HGGs occurring in infant and newborns. This latter includes 3 subgroups distinguished by molecular features: subgroup 1 involves alterations in one of the genes *ALK, ROS1, NTRK1/2/3*, or *MET;* subgroup 2, RAS/MAPK pathway alterations and hemispheric localization; subgroup 3 refers to tumors with RAS/MAPK mutations arising in midline structures (6–8). Molecular characterization of the first subgroup of this class promoted investigation of target therapies, such as Larotrectinib for NTRK-fusion positive pHGGs (NCT02576431).

**Table 1:** Pediatric-type High Grade Gliomas according to new 2021 WHO CNS classification.

Group	Molecular Features	Localization	References
*Diffuse midline glioma, H3 K27-altered*	H3 K27 mutations, EZHIP overexpression, other H3 K27 alterations.	Thalamus, brainstem, spinal cord	(4)
*Diffuse hemispheric glioma, H3 G34-mutant*	H3 G34 mutations.	Cerebral hemispheres	(4, 5)
*Diffuse pediatric-type high-grade glioma, H3-wildtype and IDH-wildtype*	Wild-type H3 and IDH gene families.	Cerebral hemispheres and midline structures	(4)
*Infant-type hemispheric glioma.*	Subgroup 1: RTK- driven Fusion genes involving ALK, ROS1, NRK and MET	Cerebral hemispheres	(4, 6–8)
	Subgroup 2: RAS/MAPK pathway mutations	Cerebral hemispheres	
	Subgroup 3: RAS/MAPK pathway mutations	Thalamus, brainstem, spinal cord	

Even though our knowledge on biological and molecular features of pHGGs largely increased during the past years, therapeutic approaches remain limited and ineffective. Current multimodal treatments encompass surgery, radiotherapy and chemotherapy, reaching 5-year survival rate less than 20% (9). New therapeutic strategies are necessary and novel immunotherapies hold great promise for possible effective treatment.

## CAR-T Cells

The principle of immunotherapy relies on restoring the physiological ability of immune system to recognize and eliminate tumor cells. This goal can be achieved through a wide variety of approaches and, so far, development of chimeric antigen receptor (CAR) expressing T cells is one of the ultimate advances in this field. CAR-T cells are T lymphocytes genetically modified by either viral vectors (retroviral or lentiviral) or by non-viral approaches (sleeping beauty transposition) to express a chimeric construct deriving from the fusion of the variable portions of a monoclonal antibody single chain to the signal transduction domains of the CD3 z chain (10). This structure combines the specificity of MHC-independent antibody recognition with the anti-tumor potential of T lymphocytes, thus allowing to transfer any antigenic specificity to T cells. In order to potentiate the antitumor efficacy of these constructs, second-generation CAR-T cells have been created by the inclusion of one costimulatory domain, such as the CD28, 41BB or OX40 molecules, resulting in a higher capacity for cytokine production, a greater expansion and a longer persistence (11). Subsequently, the combination of two signal domains into third-generation CAR-T constructs showed a further increase in the activation profile (12). CAR-T therapy has given outstanding results against several B-cell malignancies and myeloma, in both adults and children. Currently, the clinical trials reported so far on CAR-T cells directed towards the CD19 antigen, widely expressed by acute lymphoblastic leukemia cells (ALL), has documented a strong tumor activity (13, 14) even in patients highly resistant to conventional treatments or relapsed after allogeneic transplantation, obtaining CR rates of approximately 80%. Unfortunately, the results obtained so far with CAR-T cells in solid tumors have been less effective and fewer clinical trials or case reports have been reported in the literature. Several limits can hinder the development of CAR-T in solid tumors, including: the difficulty of finding a suitable target antigen (the so-called “antigen dilemma”), the strongly immunosuppressive TME, the limited persistence *in vivo* and finally, the insufficient T cell trafficking and homing to tumors and the limited persistence of exhausted T cells.

## Actual and Promising Target Antigens in pHGGs

The first antigens proposed for targeting pHGGs derive from studies conducted on adult gliomas, in particular HER-2, EphA2 and IL-13Rα2 (15).

HER-2 is a tyrosine kinase receptor overexpressed in HGGs, whose levels of expression correlate to poor outcome not only in GBM but also in other pediatric tumors, such as medulloblastoma (16, 17). HER-2 is detected at low levels in normal, healthy brain whereas is overexpressed on CNS cancer stem cells, making it an effective and safe target for the treatment of HGGs (18). Monoclonal antibodies (moAbs) recognizing HER-2 showed extraordinary results in the treatment of breast cancer, but the presence of blood brain barrier (BBB) limits their use for brain tumors. Differently from moAbs, T-cells can cross the BBB and traffic to brain tissue and cerebrospinal fluid from blood flow to recognize tumor cells, as demonstrated by clinical response of melanoma brain metastasis after intravenous infusion of adoptive tumor-infiltrating lymphocytes and by the detection of CD19-CAR-T in cerebrospinal fluids of patients with ALL (19, 20). Indeed, Ahmed et al. tested the safety of intravenous injection of virus-specific (VS) CAR-T cells directed to HER-2 in a phase I clinical trial on patients affected by high grade gliomas: with 17 patients treated, including 7 children <18 years, the approach resulted to be safe, without any dose-limiting toxicity (DLT) reported (21). Interestingly, one of the adolescent patients with unresectable right thalamic HGG showed the reduction of 30% of the longest tumor diameter lasted for 9.2 months and an Overall Survival (OS) of 34.2 months after two infusions of CAR-T cells (21). In order to target CNS tumors, CAR-T can be delivered locally in tumor resection cavity or in the ventricular system with improved tumor control, even at lower doses, and reduced systemic circulation and toxicity, as recently highlighted by Theruvat et al. and Donovan et al. (22–24). Currently, two clinical trials are evaluating intracranial injection of Anti-HER-2 CAR-T for treatment of pHGGs (NCT02442297; NCT03500991) (**Table 2**). Preliminary results of the phase 1 trial “BrainChild-01” showed that repetitive intracranial infusion of HER-2 CAR-T on a cohort of pediatric and young adult patients with CNS tumors is safe, well tolerated, and able to induce immune response (18). Two other clinical trials, namely “Brainchild-02” and “BrainChild-03”, are evaluating the effectiveness of locoregional infusion of CAR-T cells targeting EGFR and B7-H3, respectively (NCT03638167 NCT04185038).

**Table 2:** Ongoing clinical trials evaluating CAR-T therapies for pHGGs.

Official title	Antigenic target	Administration	Responsible party	NCT
T Cells Expressing HER2-specific Chimeric Antigen Receptors (CAR) for Patients With HER2-Positive CNS Tumors (iCAR)	HER-2	Locoregional delivery	Nabil Ahmed, Baylor College of Medicine	02442297
HER2-specific CAR-T Cell Locoregional Immunotherapy for HER2-positive Recurrent/Refractory Pediatric CNS Tumors	HER-2	Locoregional delivery	Julie Park, Seattle Children’s Hospital	03500991
Genetically Modified T-cells in Treating Patients With Recurrent or Refractory Malignant Glioma	IL13Rα2	Locoregional delivery	City of Hope Medical Center	02208362
EGFR806-specific CAR-T Cell Locoregional Immunotherapy for EGFR-positive Recurrent or Refractory Pediatric CNS Tumors	EGFR	Locoregional delivery	Julie Park, Seattle Children’s Hospital	03638167
GD2 CAR-T Cells in Diffuse Intrinsic Pontine Gliomas (DIPG) & Spinal Diffuse Midline Glioma(DMG)	GD-2	Intravenous injection after Lymphodepletion with Cyclophosphamide/Fludarabine Chemotherapy	Crystal Mackall, Stanford University	04196413
C7R-GD2.CAR-T Cells for Patients With GD2-expressing Brain Tumors (GAIL-B)	GD-2	Intravenous injection Lymphodepletion with Cyclophosphamide/Fludarabine Chemotherapy	Bilal Omer, Baylor College of Medicine	04099797
Study of B7-H3-Specific CAR-T Cell Locoregional Immunotherapy for Diffuse Intrinsic Pontine Glioma/Diffuse Midline Glioma and Recurrent or Refractory Pediatric Central Nervous System Tumors	B7-H3	Locoregional delivery	Julie Park, Seattle Children’s Hospital	04185038
B7-H3-Specific Chimeric Antigen Receptor Autologous T-Cell Therapy for Pediatric Patients With Solid Tumors (3CAR)	B7-H3	Intravenous injection Lymphodepletion with Cyclophosphamide/Fludarabine Chemotherapy	St. Jude Children’s Research Hospital	04897321

EphA2 is a tyrosine kinase receptor involved in oncogenic pathways in several tumors, including breast cancer, lung cancer and HGG (25). High expression of EphA2 has been correlated to worse clinical outcomes in adult HGG and recently the same association was confirmed in pediatric HGGs (26). Preclinical studies reported a promising antitumoral activity of EphA2-redirected CAR-T-cells for the treatment of HGG. Recently, results of the first-in-human trial of intravenous administration EphA2-CAR-T in patients with high grade gliomas showed safety and transient clinical responses (27). However, the trial enrolled patients >18 years, therefore the efficacy of EphA2- CAR-T in pediatric cohorts remains to be investigated.

IL13Rα2 is a monomeric, high-affinity, IL13 receptor, detected at high levels in more than 50% of HGGs, whose overexpression is associated with poor outcomes (28). Brown et al. started a clinical trial to evaluate the efficacy of intracranial infusion of IL13Rα2-CAR-T in adult and pediatric patients with HGGs (NCT02208362). The first published results show safety of the therapeutic approach and documented a complete radiographic response in a patient for up to 7.5 months (29).

Recently, both GD2 and B7-H3 were found to be highly expressed in pediatric diffuse midline glioma (DIPG). Interestingly, Haydar et al. established a hierarchy of antigens expressions in pediatric brain tumors showing that, despite a high heterogeneity, GD2 and B7-H3 maintain the highest expression as compared to IL-13Rα2, HER2 and EphA2 (30). These data suggest the importance of focusing on these targets for pHGGs rather than antigens mostly relevant in adult gliomas.

GD2 is involved in mechanisms of cell growth, motility and invasiveness of several pediatric tumors like neuroblastoma, Ewing’s Sarcoma and pHGGs (31). After the promising results of clinical trials using several generations of GD2-CAR-T cells to treat patients affected by neuroblastoma, first, and other GD2^+^ solid tumors, after, (NCT03373097; NCT04099797) this approach is currently under preclinical and clinical evaluation also for pHGG (32, 33); NCT05298995). In particular, Mount and Majzner et al. demonstrated uniform and high levels of GD2 on H3-K27 diffuse midline glioma cells and developed a second-generation GD2 CAR construct which showed efficient anti-tumor activity in DMG PDXs models (34), leading to the activation of a phase I clinical trial, currently enrolling pediatric patients. They published the results of the first 4 patients with H3K27-altered diffuse midline glioma (DMG) treated in the trial and showed that the toxicity profile strongly depends on the tumor location and is manageable and reversible with intensive supportive care (35). In addition, reduction of neurological deficits and radiological improvement after CAR-T administration were reported. In particular, in one of the treated patients, affected by spinal cord DMG, a reduction of 90% of the tumor volume after the first intravenous administration and a further reduction of neoplasm dimensions of 80% after a second intraventricular injection was observed (34). At Bambino Gesù Children’s Hospital we are currently activating a phase I clinical trial to test the safety and, preliminarily, efficacy of third-generation (incorporating 4.1BB and CD28 costimulatory domains) GD2-CAR T cells, infused i.v., for the treatment of pediatric patients affected by CNS tumors (36); NCT05298995). In particular, in view of the correlation between tumor location and toxicity, the study has an innovative design of sequential enrollment of patients into 3 different arms, the first being represented by medulloblastoma and other embryonal tumor (arm A), the second by hemispheric HGG (arm B) and the third by tumors with midline location, at higher risk of severe toxicity, namely thalamic HGG, DMG, DIPG (arm C).

B7-H3 is a transmembrane protein with important immune inhibition functions, belonging to the B7 family of immune checkpoint proteins, and is overexpressed in several pediatric malignancies - including pHGGs (37). Majzner et al. tested the efficacy of B7-H3 CAR-T in xenografts models of several different paediatric solid tumors, including osteosarcoma, medulloblastoma and Ewing’s sarcoma) (38). Interestingly, they showed a potent antitumor activity, strictly dependent on the antigen density on tumors, which resulted to be aberrantly high compared to healthy tissues (38). The results is extremely important, considering the wide expression of the antigen in normal tissues. Recently, phase 1 clinical trials exploring the safety of either loco-regional or intravenous infusion of B7-H3 CAR-T cells for the treatment of paediatric patients with solid tumors, including pHGGs, started (NCT04185038; NCT04897321).

As already mentioned, the success of CAR-T cell therapy relies on the choice of target antigens highly expressed on tumor cells, amongst several factors. However, the recognition of a single antigen is limited by the possible occurrence of the so-called “antigen escape” phenomenon: either the down-regulation of the target antigen or the selection of already negative subclones by the pressure of the treatment, in highly heterogeneous tumors, can represent the cause of subsequent tumor recurrence. This issue is particularly relevant in the setting of pHGG which are extremely heterogeneous and often characterized by subpopulations of tumor cells with different antigen expression. For this reason, CAR-T cells able to recognize multiple antigens on target cells by incorporating 2 antigen targeting domains within one CAR construct have been developed. For HGG, a tandem CAR (TanCAR) targeting simultaneously HER-2 and IL-13Rα2 was designed (39). TanCAR-T cells showed an elevated capacity to lyse glioma cells *in vitro* and *in vivo* and to prevent tumor recurrence in the animal model. Moreover, CAR-T cells targeting 3 glioblastoma associated antigens (HER2, IL13Rα2, and EphA2) were also developed and showed even more effectiveness as compared to the bispecific CAR-T (40). Another interesting strategy to overcome antigenic escape in HGG is the combination of CAR-T cells with Bi-specific T-cell engagers (BiTEs) targeting different antigens. Choi et al. developed EGFRvIII-redirected CAR-T cells secreting EGFR-BiTEs in the tumor site (41). This strategy was tested in mouse models and resulted capable of dulling the antigenic loss effects. Moreover,- since systemic administration of EGFR-BiTE could induce off-tumor toxicities - the local release by these CAR-T cells reduces the risk of cytotoxic T-cells activity on healthy tissues expressing EGFR.

## Tumor Microenvironment in Adult and Pediatric High-Grade Glioma

The complex intra- and inter-tumor heterogeneity of the tumor microenvironment (TME) plays a crucial role in mediating tumor progression and resistance to therapy (42). Several different cells, including tumor-associated glioma stem cells (GSC), stromal cells (resident brain glial cells, oligodendrocytes, astrocytes, ependymal cells, microglia) and infiltrating immune cells, are key regulators of growth and vascularization of HGGs (43, 44).

In adult HGGs, TME presents a wide range of immune suppressive mechanisms preventing tumor recognition and eradication by the innate and adaptive immune system. The presence of immuno-suppressive cytokines (TGF-β, IL-10), chemokines, and regulatory immune-suppressive cells, such as tumor-associated macrophages/microglia (GAMs) and myeloid-derived suppressor cells (MDSCs), limits the effectiveness of current therapies and are briefly review below (45–47).

GSCs are characterized by their self-renewal properties and play a key role in drug escape mechanisms, for example driving radiation-induced DNA methylation changes after radiotherapy (48) GSCs can promote a microinvasion of healthy tissues in areas that support their proliferation – such as subventricular, perinecrotic and perivascular areas (49) - evading therapeutic agents and contributing to recurrence of disease. Recently, a mouse model of disease derived by orthotopic transplantation of GSCs of pHGGs samples was developed (50). Methylation, histology and clinical outcomes resembled accurately the patients’ tumors of origin, indicating the ability of GSCs to differentiate in tumor cells. One of the most innovative approaches targeting GSCs niche in pHGGs is represented by immunovirotherapy (51). Friedman et al. observed the ability of oncolytic HSV-1 to kill tumor cells and CD133+ GSCs in preclinical models (52). Results from a phase 1 clinical trial on the use of oncolytic HSV1 in patients with pHGGs indicated the safety of this approach (53). Combining T-cells based therapies with this kind of modern immunological approaches affecting GSCs could significantly improve tumor control.

GAMs represent an important component of the glioma microenvironment (54) constituting the main proportion of infiltrating cells in adult and pediatric HGGs (i.e., glioblastoma and DIPG) (55, 56). Inside the tumor, GAMs usually acquire a specific phenotype of activation that favors tumor growth, angiogenesis and promotes the invasion of normal brain parenchyma. Most of the macrophages recruited into TME, polarize toward an M2-like phenotype and exhibit suppression of the proliferation and functionality of tumor-infiltrating T cells through the production of anti-inflammatory cytokines (57). In adult HGG, GAMs have been extensively described as pivotal drivers of progression and survival of malignant cells in the TME (58). Hence, several anti-GAMs aproaches are currently under evaluation for treatment of adult GBM. Recently, Lin et al. studied TME and GAMs features specifically in DIPG (59). Although DIPG tumor cells produce Colony Stimulating Factor 1 (CSF1), a cytokine associated with the M2 pro-tumorigenic phenotype, DIPG-associated macrophages do not seem to have the characteristic of macrophages of type 2 (56, 60). Transcriptome analysis have shown that GAMs from DIPG express lower levels of some inflammatory cytokines and chemokines (IL6, IL1A, IL1B, CCL3, CCL4) compared to adult HGG-associated macrophages. Moreover, Engler et al. observed a negative correlation between levels of GAMs accumulation and survival rate in adult HGGs but not in pediatric tumors, highlighting the marked biological differences between the pediatric and adult counterpart (59). In addition, the analysis of lymphocyte infiltrate in primary DIPG tissues reveals the lack of tumor-infiltrating lymphocytes (TILs), defining it as a immunologically “cold” tumor (56). For this reason, adoptive immunotherapies (e.g. CAR-T cells) leading to the transferal of T-cells in the TME represents a promising strategy to induce a strong inflammatory and antitumor response.

MDSCs are involved in immune suppression in several types of cancers, including HGG (61). Although only few studies focus on their role specifically in pHGGs, recently Mueller et al. reported a correlation between high levels of circulating MDSCs and poor prognosis in patients with DIPG, suggesting a role in immunosuppression and tumor escape mechanisms in pHGGs as well (62). Moreover, MDSCs showed to impair efficacy of immunotherapy in other pediatric tumors such as neuroblastoma, representing a relevant target to improve efficacy of modern CAR-T cells therapies (63).

Successful immune escape of tumor cells includes also the production of soluble factors in the microenvironment by tumor cells (TGF-β, LDH5), the induction of co-inhibitory molecules (PD-1, LAG-3 and TIM-3) and the release of immunosuppressive factors (CSF-1, VEGF, PGE2, NO, Arg I, IDO and Gal-1) (64). In adult HGG the production of lactate dehydrogenase isoform 5 (LDH5) and TGF-β impairs NK cells cytotoxic function, usually relevant for the elimination of glioma cells (65). Although in adult HGGs there is a strong infiltration of NK and myeloid cells into the tumor, a similar finding in pediatric brain tumors has not been reported (66).

## Next Generation CAR-T Cells and Combined Strategies

Solid tumor, including pHGG, showed important mechanisms of resistance to current CAR-T cell therapies. Important obstacles to CAR-T efficacy is the presence of immunosuppressive factors in TME – including, but not limited to, TGF-β, PD-1 or CTLA4 mediated signals (67, 68) – which prevent CAR-T cells expansion and finally induce their exhaustion (69). For this reason, strategies able to circumvent these barriers are needed to improve the effectiveness of CAR-T therapies for pHGGs.

**Figure 1:**
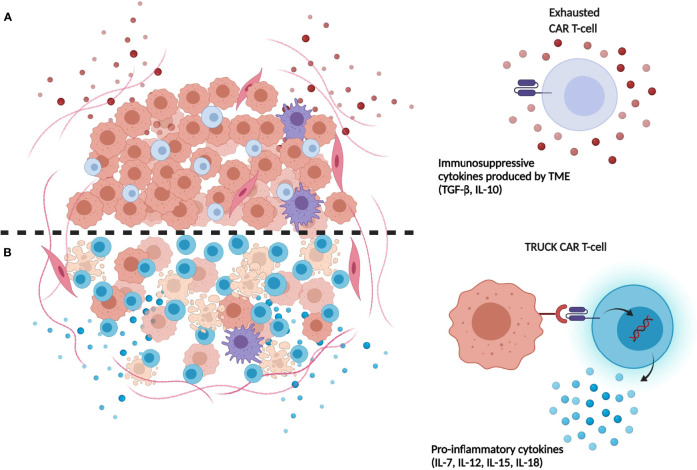
Limitations of first-generation CAR-T-cells compared to next generation TRUCK CAR-T cells. **(A)** Immunosuppressive cytokines (e.g. TGF-β, IL-10) released in TME by tumor cells induce repression and exhaustion of CAR-T cells. **(B)** TRUCK CAR-T cells release transgenic immunostimulatory cytokines which promote their resistance and expansion in tumor site, contrasting TME immunosuppression mechanisms. (Illustration created with BioRender.com).

Recently, CAR-T cells releasing transgenic cytokines after activation at tumor site have been developed. This approach was conceived to overcome the insufficient production of pro-inflammatory cytokines by T cells accumulated in the tumor environment. In details, T-cells redirected for universal cytokine-mediated killing (TRUCKs) cells are fourth generation CAR-T cells, armed with immune stimulatory cytokines that improve CAR-T cell expansion and persistence (70) **(** **Figure 1**). In details, CAR-T cells can be engineered with inducible expression cassette for the cytokine of interest, including IL-7, CCL-19, IL-15, IL-18, and IL-1 (71–73). In particular, transgenic expression of IL-15 could be an appealing strategy to enhance CAR-T cell effector function in HGGs patients, thanks to the well-known ability of this cytokine to induce a more memory stem cell-like phenotype of transduced T cells (73). The group of Krenciute et al. for example, showed an increased persistence and a greater antitumor activity of IL-13Rα2-CAR-T cells expressing IL-15 constitutively as compared to conventional IL-13Rα2-CAR-T cells (74, 75). Also, anti-GD2 TRUCKs secreting IL-12 or IL-18 after activation have been developed, showing improved T-cells activation and increased monocyte recruitment after *in vitro* migration assay (76). On one hand, cytokines release following CAR-T-cells activation results in improved CAR-T cell persistence and stronger antitumor activity, both *in vitro* and *in vivo*. On the other hand, continuous release of secreted cytokine could cause toxicities, limiting the clinical therapeutic window of these CAR-T cells (74). This limitation could be overcome by the development of CAR-T cells expressing constitutively active cytokine receptors, such as IL-2, IL-7, and IL-15 receptors, able to activate the relative intracellular axis, instead of releasing the soluble cytokines. In particular, the expression of constitutively signaling IL-7 receptor (C7R) on a EphA2-CAR-T produced promising results in terms of proliferation, survival and antitumoral activity of CAR-T cells both *in vitro* and in orthotopic xenograft models of HGG (77).

Another major obstacle provided by the TME, to the activation and expansion of CAR-T cells is represented by the widely expressed immune checkpoint receptors – such as PD-1, CTLA-4, TIM-3, and LAG-3. Conventional immune checkpoint inhibitors (ICIs) blocking CTLA-4 (i.e. ipilimumab) or PD-1 (i.e. nivolumab) have shown great success in some solid tumors, including non-small cell lung cancer and metastatic melanoma, but not in HGGs, probably owing to the negligible infiltration of effector T cells in these tumors and to the low mutational burden of pHGGs, leading to few immunogenic tumor neoantigens (78–80). Nevertheless, combined with CART cells, ICIs might improve the ability of the transgenic T cells to exert their antitumor activity, overcoming the exhaustion induced by the TME (81). In pre-clinical models of glioma, checkpoint blockade has been studied as an adjuvant to improve the efficacy of CAR-T therapy. For example, combination of HER-2 redirected CAR-T cells with anti-PD1 antibody induced enhancement of CAR-T cells activity against HGG cells *in vitro* (82). Furthermore, Song et al. reported the ability of anti-PD1 antibodies to improve EGFRvIII-CAR-T cells antitumoral effects in a mouse model of HGG (83), suggesting that PD1 blockade might represent an effective strategy. Based on these promising pre-clinical studies, two phase I clinical trials are currently investigating 2^nd^ generation CAR-T cells in combination with pembrolizumab or nivolumab for the treatment of adult patients with HGG (NCT03726515; NCT04003649).

Interestingly, the role of some intracellular signaling pathways in the activity of CAR-T cells has been investigated, unveiling new potential approaches to improve CAR-T cells efficacy. For example, the diacylglycerol kinase (DGK), a physiologic negative regulator of the signal transduction of the T-cell receptor (TCR), is able to negatively regulate CAR-T cell activation (84). Therefore, the knockout of DGK can induce an improvement of the anti-tumor cytotoxicity of CAR-T cells. In a mouse glioma model, EGFRvIII-CAR-T cells lacking DGK revealed elevated effector function of the transgenic CAR-T cells, with increased antitumor activity and tumor infiltration (85). Moreover, recently, we reported that the co-administration of linsitinib – a dual IGF1R/IR inhibitor – is able to improve GD2-CAR-T antitumor activity and increase tumor cell death of primary cells of H3K27 diffuse midline glioma, both *in vitro* and *in vivo* (86). These results support the hypothesis that the use of combinatorial approaches might potentiate the efficacy of CAR-T cells for the treatment of pHGGs.

Another promising and sophisticated immunotherapy approach is represented by oncolytic viruses (OV): genetically modified viral agents able to replicate in tumor cells with a negligible replication ability in non-neoplastic cells (87). OV antitumoral activity is based on two mechanisms: i) induction of direct lysis of tumor cells through infection and replication; ii) stimulation of effector function and antitumor activity of the T cells in TME. The latter mechanism as shown to be extremely relevant, if not even the most relevant, in the antitumor activity of OV and represents the rational for combining OV and CAR-T cells for the treatment of solid tumors, with the aim to increase trafficking and antitumoral cytotoxicity of T-cells in TME (88). In particular, Huang et al. studied a preclinical model of the combination of anti-B7-H3 CAR-T cells with and IL-7-loaded oncolytic adenovirus (oAD-IL7) for the treatment of HGG (89). The combined strategy promoted T-cells proliferation and antitumoral activity *in vitro* and reduced mortality in the xenograft mouse models (89). Conversely, there are some controversial results showing the inefficacy of OVs and CAR-T cells combination for HGGs. Recently, it was observed that the pro-inflammatory activity of an OV (VSVmIFNβ) can impair EGFRvIII CAR-T cells cytotoxicity against HGG cells (90). The reason of these unexpected results may lie on the complexity of inflammation mechanisms occurring in TME, which need to be better understood in order to develop precise, effective, and safe combination strategies involving CAR-T cells. Moreover, the group of Park et al. developed an interesting combined strategy exploiting OVs to induce the expression of the CAR target in infected tumor cells, hence increasing CAR-T cells antitumoral activity (91). In details, in this approach an engineered OV delivers a transgene leading to the expression of a truncated form of CD19 (CD19t) on the neoplastic cells, promoting the cytotoxic activity of CD19-CAR-T cells.

Lastly, reduced T-Cell trafficking and homing in the TME was underlined as one of the major obstacles of CAR-T therapies. In particular, reduced production of chemokines and modification of Extra-Cellular Matrix (ECM) are involved in hindering the migration of T-cell to the tumor site (92). Interestingly, Jin et al. developed CAR-T cells expressing chemokines receptors (CXCR1 and CXCR2) to improve intratumoral trafficking (93). The results observed in xenograft models of HGG confirmed the efficacy of this approach, unveiling the importance of increased T-cells homing to improve CAR-T therapies efficacy. Moreover, the CNS location adds a relevant and peculiar obstacle to the migration of CAR T cells to tumor: the presence of the BBB, a permeability barrier characterized by the connection, through tight junctions, of endothelial cells with the luminal and abluminal membranes lining the capillaries of the brain. Despite the documented ability of i.v. administered CAR T-cells to cross the BBB, as already mentioned, targeted delivery of T cells at the level of the CNS is an attractive option to reduce systemic toxicity and increase CAR T-cell concentration at tumor site. Indeed, as mentioned above, a superior efficacy with reduced toxicity of intraventricular/intrathecal administration of CAR-T cells was already shown (23, 29) and the strategy represents a valid and promising approach to circumvent the BBB obstacle.

Despite all the presented approaches show great potential to improve CAR-T cell function and safety in preclinical models, their use in clinical setting is limited at present. The results of future clinical trials will shed new lights on potential and limitations of these highly innovative approaches.

## Conclusions and Future Strategies

Conventional therapies, radiation and chemotherapy are not sufficient for achieving a sustained disease remission in patients affected by HGG, both in adult that pediatric patients, and new therapeutic strategies are necessary. Immunotherapy is a new therapeutic approach that harnesses the inherent activity of the immune system to control and eliminate malignant cells. To date, CAR-T cell therapy has shown promise in early clinical trials in HGG patients but could not achieve the same sustained success observed in hematological malignancies. Several hurdles, including the immunosuppressive TME, the heterogeneity in target antigen expression and the difficulty of accessing the tumor site, impair the antitumor efficacy of CAR-T cells. Several CAR-T antigenic targets have been considered so far, and recently GD2 and B7-H3 look very promising for pediatric tumors. However, heterogeneity of expression is a limiting factor for single antigen-redirected CAR-T in solid tumors. For this reason, considering innovative strategies such as next generation CAR-T cells or combinatory approaches with other immunotherapy agents (e.g. BiTEs, Oncolytic viruses and ICIs) could improve tumor control. “Next generation” or multivalent CAR-T have been developed and might have a large impact on treatment of pHGG, improving efficacy of T cells therapies and overcoming obstacles of TME.

## Author Contributions

LA, GC, and FB conceived the article. LA and GC compiled the review and prepared the draft of the manuscript. FB, AM, AC, and GB critically reviewed the manuscript. FB edited the manuscript. All authors contributed to the article and approved the submitted version.

## Funding

The work was partly supported by grants from: AIRC (Associazione Italiana Ricerca sul Cancro, My First AIRC – ID 20450 - to FB) and Ministero della Salute (Ricerca Corrente and 5x1000 to FB).

## Conflict of Interest

The authors declare that the research was conducted in the absence of any commercial or financial relationships that could be construed as a potential conflict of interest.

## Publisher’s Note

All claims expressed in this article are solely those of the authors and do not necessarily represent those of their affiliated organizations, or those of the publisher, the editors and the reviewers. Any product that may be evaluated in this article, or claim that may be made by its manufacturer, is not guaranteed or endorsed by the publisher.
